# Discourje: Runtime Verification of Communication Protocols in Clojure

**DOI:** 10.1007/978-3-030-45190-5_15

**Published:** 2020-03-13

**Authors:** Ruben Hamers, Sung-Shik Jongmans

**Affiliations:** 8 Johannes Kepler University, Linz, Austria; 9 University of Birmingham, Birmingham, UK; 10 Open University, Heerlen, the Netherlands; 11 CWI, Amsterdam, the Netherlands

## Abstract

This paper presents Discourje: a runtime verification framework for communication protocols in Clojure. Discourje guarantees safety of protocol implementations relative to specifications, based on an expressive new version of multiparty session types. The framework has a formal foundation and is itself implemented in Clojure to offer a seamless specification–implementation experience. Benchmarks show Discourje’s overhead can be less than 5% for real/existing concurrent programs.

### 1 Introduction

**Background.** To take advantage of today's and tomorrow's multi-core processors, shared-memory concurrent programming—a notoriously complex enterprise—is becoming increasingly important. To alleviate some of the complexities, in addition to low-level *synchronization primitives*, several modern programming languages have started to offer core support for higher-level *communication primitives* as well, in the guise of message passing through *channels* (e.g., Go [25], Rust [42], Clojure [17]). The idea is that, beyond their usage in distributed computing, channels can also serve as a *programming abstraction* for shared memory, supposedly less prone to concurrency bugs than locks, semaphores, and the like. However, in a recent study of 171 concurrency bugs in popular open source Go programs [48], Tu et al. found that "message passing does not necessarily make multi-threaded programs less error-prone than shared memory."

From a programmer's perspective, a key problem is this: if we already know which *roles* (threads), *infrastructure* (channels between threads), and *protocols* (communications through channels) our program should consist of, then how can we ensure our implementation is indeed *safe* relative to our specification? Safety means "bad" channel actions never occur: <u>if</u> a send, receive, or close happens in the implementation, <u>then</u> it is allowed by the protocol in the specification. For instance, typical protocols rule out common message-passing concurrency bugs [48], such as sends without receives, receives without sends, and type mismatches (actual type sent $\neq$ expected type received). Essentially, thus, we face a classical verification problem, with classical ingredients: an implementation language $\mathcal{I}$, a specification language $\mathcal{S}$, and an inclusion relation $\preceq$.

Over the past years, a significant body of research in this area has been based on *multiparty session types* (MPST) [27]. The idea is to specify protocols as behavioral types [1,30] against which threads are subsequently type-checked; the

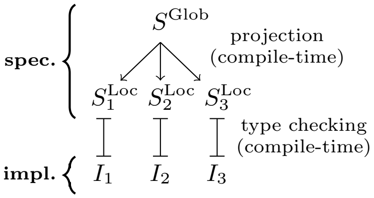

**Fig. 1.** MPST

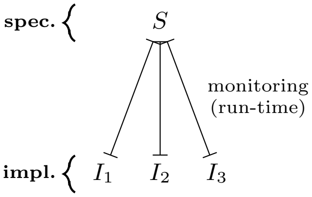

**Fig. 2.** This paper theory guarantees that static well-typedness of threads at compile-time implies dynamic safety of their channel actions at run-time. Originally [27], $\mathcal{I}$ was a dialect of pi-calculus, $\mathcal{S}$ was a calculus of behavioral types, and $\preceq$ was defined through formal typing rules, but more recently, practical implementations were developed as well [14,28,29,37,38,44], where $\mathcal{I}$ is an existing *general-purpose language* (GPL; Erlang, F#, Go, Java, Scala), $\mathcal{S}$ is a new *domain-specific language* (DSL; Scribble), and $\preceq$ encodes behavioral types in $\mathcal{S}$ as non-behavioral types in $\mathcal{I}$ (e.g., through custom communication API generation [29]). These works highlight two key strengths of the MPST methodology, namely it supports:

##1 fully automated verification of *concrete programs* (vs. abstract models);
#2 user-friendly *programming language-based notation* to write specifications of protocols (vs. dynamic logic or temporal logic).

**Problem.** One of the key open problems of MPST concerns *expressiveness*. For instance, suppose we need to write a program in which messages are repeatedly communicated from threads $I_1$ and $I_2$ to thread $I_3$, non-deterministically ordered (i.e., standard producers–consumer); this protocol is not supported by MPST.

We identify two reasons why expressiveness is limited.

**First**, MPST were originally developed for distributed computing (service choreographies [10,11]); accordingly, *decoupled* verification of roles (per-service type-checking) has always been a key requirement [14]. This is reflected in the MPST workflow (Fig. 1): first, the programmer writes a *global* protocol specification; then, an MPST tool *projects* it onto every role to infer *local* protocol specifications; then, the implemented threads are type-checked. However, role-based decomposition of global behavior into equivalent local behaviors often cannot be done statically (e.g., [12]), so expressiveness is limited by "projectability".

**Second**, MPST prescribes static type-checking, which limits expressiveness, because: (a) type-checking is sound, but not complete, so the static MPST approach rejects implementations that are conservatively ill-typed but actually safe; (b) protocols whose execution relies on value-dependent control flow are supported only in limited circumstances. To alleviate (b), value-dependent type constructors can be added to $\mathcal{S}$ [20,47], but this raises practical issues (i.e., dependent types are only scarcely supported by mainstream GPLs).

**Contributions.** To simplify shared-memory concurrent programming in languages with channels, we aim to consolidate strengths #1 and #2 (page 267), but alleviate MPST's expressiveness issues. Specifically, this paper is founded on two tenets that depart from existing work in significant ways (Fig. 2).

**First**, we exploit the fact that in our context, channels serve "merely" as programming abstractions for shared memory; there is no distribution whatsoever. Thus, whereas MPST-based verification for distributed computing requires projection, this is not the case in our setting, opening the door to fully automated *projection-free* MPST and eliminating a significant source of restrictions.

**Second**, instead of adopting MPST-based verification through static typechecking at compile-time, we explore MPST-based verification through *dynamic monitoring at run-time*. This enables soundness *and completeness*, while it also supports *value-dependent protocols* in a generally implementable way (i.e., we are not aware of a mainstream GPL that does not support our monitoring approach).

In this paper, we present our practical embodiment of these ideas: *Discourje* (pronounced "discourse"), a runtime verification framework for communication protocols in *Clojure* [17,26]. Discourje consists of two components: a DSL to specify protocols and construct monitors, and an API to implement protocols (supplementing Clojure) and add instrumentation. While we could have developed this framework for any language with channel-based programming abstractions, including Go and Rust, Clojure is particularly interesting, because: (1) Clojure has a powerful macro system that enabled us to develop the Discourje DSL as an extension to Clojure, thereby offering programmers a seamless specification–implementation experience; (2) contrasting Go and Rust, Clojure is not a systems language but an applications language, so runtime verification overheads might be more tolerable. We summarize our contributions as follows:

- **Overview** (Sect. 2): Discourje guarantees *safety* of protocol implementations, it provides *freedom from data races* in pure Clojure, and it is *more expressive* than existing MPST tools, as demonstrated through examples.
- **Design** (Sect. 3): We developed *core calculi*, including operational semantics, for Clojure and the Discourje DSL as a theoretical foundation.
- **Implementation** (Sect. 4): We implemented Discourje fully in Clojure. The Discourje DSL comprises Clojure *macros*, while the Discourje API is a wrapper around Clojure functions to add instrumentation, *non-invasively.*
- **Evaluation** (Sect. 5): Through benchmarks, we show that Discourje's overhead can be less than 5% for real/existing concurrent programs.

Our artifact is available at https://github.com/discourje.

### 2 Overview

**Clojure (in a nutshell).** Clojure [17,26] is a general-purpose, impure functional language that compiles to Java bytecode. As a dialect of Lisp, Clojure follows the code-as-data philosophy, provides a powerful macro system, and adopts parenthesized prefix notation. Clojure offers asynchronous channel-based programming abstractions through core library `clojure.core.async` [16]. In the annual Clojure survey [15], Clojure programmers indicate "ease of development" is more important than "runtime performance"; this makes Clojure an interesting target for runtime verification (viz. overheads).

To introduce the core features of Clojure relevant to this paper, Fig. 3 shows a channel-based concurrent Tic-Tac-Toe program in Clojure[3] while Fig. 4 summarizes the meaning of every primitive (";;" indicates comments). Lines 1–9 define constants (`blank`, `cross`, `nought`, `initial-grid`) and functions (`get-blank`, `add`, `not-final?`) to represent Tic-Tac-Toe concepts. Lines 11-12 define two channels (`a->b` and `b->a`) that implement the infrastructure through which players Alice and Bob communicate. Channels in Clojure are bounded: sends/receives block until a channel is not full/empty. Lines 14–24 and 25–35 define threads that implement Alice and Bob. Both players execute a loop, starting with a blank grid. In each iteration, Alice first gets the index of some blank space on the grid, then plays a cross in that space, then sends a message to Bob to communicate the index, then awaits a message from Bob, and then updates the grid accordingly; Bob acts symmetrically. After every grid update, Alice or Bob checks if it has reached a final configuration; if so, the loop is exited and channels are closed.

Every Clojure data structure, including the vector that implements the grid, is *persistent*, and therefore, effectively *immutable*. This means that every operation on an existing data structure leaves it intact, and instead, it returns a new data structure. Thus, Alice and Bob initially share the same initial grid, but because it cannot be modified in-place, modifications need to be explicitly communicated. Persistence of Clojure data structures is also why we can guarantee freedom from data races in pure Clojure (= Clojure without Java objects): if users communicate only Clojure data through channels, race freedom is guaranteed (if Java objects are communicated, the user is responsible to avoid races).

**Basic Discourje: Tic-Tac-Toe.** A basic Discourje specification of the Tic-Tac-Toe protocol for Alice and Bob is shown in Fig. 5. We typeset Discourje "keywords" (which are actually just Clojure functions and macros) **`bold violet`**.

Lines 1–2 define two roles (**`role`**) to represent Alice and Bob. Lines 4–6 define an auxiliary specification, inserted twice into the main specification (**`ins`**); it states that the channels between Alice and Bob are closed (**`-##`**), in parallel (**`par`**). Lines 7–13 define the main specification; it states that recursively (**`fix`**), first a message of type `Long` (the index of a grid) is communicated from Alice to Bob (**`-->`**), and then from Bob to Alice, unless the channels are closed (the game is done). Square brackets are used to build lists of sub-specifications (sequencing).

The Tic-Tac-Toe protocol depends on value-dependent control flow, as Alice and Bob close the channels only once the grid has reached a final configuration. This is a non-protocol-related property that no existing MPST tool supports.

[3] Tic-Tac-Toe is a two-player game played on a 3x3 grid. Players take turns to fill the initially blank spaces of the grid with crosses ("X") and noughts ("O"). The first player to fill three adjacent spaces, in any direction, with the same symbol wins.

```
(def blank " ") (def cross "x") (def nought "o")

(def initial-grid [blank blank blank   ;; an initial 3x3 grid of blank spaces,
                   blank blank blank   ;;   implemented as a vector of length 9
                   blank blank blank]) ;;   (persistent data structure)

(def get-blank  (fn [g]          ...)) ;; returns a blank space in g
(def add        (fn [g i x-or-o] ...)) ;; returns g, but with i set to x-or-o
(def not-final? (fn [g]          ...)) ;; returns true iff g is not final

(def a->b (chan 1)) (def b<-a a->b) ;; b<-a is an alias of a->b
(def b->a (chan 1)) (def a<-b b->a) ;; a<-b is an alias of b->a

(thread ;; alice
  (loop [g initial-grid]
    (let [i (get-blank g)
          g (set g i cross)]
      (>!! a->b i)
      (if (not-final? g)
        (let [i (<!! a<-b)
              g (set g i nought)]
          (if (not-final? g)
            (recur g))))))
  (close! a->b))

(thread ;; bob
  (loop [g initial-grid]
    (let [i (<!! b<-a)
          g (set g i cross)]
      (if (not-final? g)
        (let [i (get-blank g)
              g (set g i nought)]
          (>!! b->a i)
          (if (not-final? g)
            (recur g))))))
  (close! b->a))
```

**Fig. 3.** Clojure implementation of Tic-Tac-Toe (dashed arrows: matching send/receive)

Library `clojure.core` (basic):

- **(def** $x$ $e$**)**: first evaluates $e$ to $v$; then binds $x$ to $v$ in the global environment.
- **(fn** [$x_1$ ... $x_n$] $e_1$ ... $e_m$**)**: evaluates to a function with parameters $x_1$, ..., $x_n$ and creates a recursion point; then, when applied to arguments $v_1$, ..., $v_n$, sequentially evaluates $e_1$, ..., $e_m$ with $x_1$, ..., $x_n$ bound to $v_1$, ..., $v_n$.
- **(let** [$x_1$ $e_1$ ... $x_n$ $e_n$] $e$**)**: first evaluates $e_1$ to $v_1$; then evaluates $e_2$ to $v_2$ with $x_1$ bound to $v_1$; ...; then evaluates $e_n$ to $v_n$ with $x_1$, ..., $x_{n-1}$ bound to $v_1$, ..., $v_{n-1}$; then evaluates $e$ with $x_1$, ..., $x_n$ bound to $v_1$, ..., $v_n$.
- **(loop** [$x_1$ $e_1$ ... $x_n$ $e_n$] $e$**)**: same as **let**, but also creates a recursion point.
- **(recur** $e_1$ ... $e_n$**)**: first evaluates $e_1$, ..., $e_n$ to $v_1$, ..., $v_n$; then evaluates the nearest recursion point with $x_1$, ..., $x_n$ bound to $v_1$, ..., $v_n$.
- **(if** $e_1$ $e_2$ $e_3$**)**: first evaluates $e_1$; if `true`, evaluates $e_2$; else, evaluates $e_3$.

Library `clojure.core.async` (concurrency):

- **(>!!** $c$ $e$**)**: first evaluates $e$ to $v$; then sends $v$ through channel $c$.
- **(<!!** $c$**)**: receives a value through channel $c$.
- **(close!** $c$**)**: closes channel $c$.
- **(chan** $n$**)**: evaluates to a channel with a buffer of size $n$.
- **(thread** $e$**)**: creates a new thread that evaluates $e$

**Fig. 4.** Clojure primitives

```
(def alice (role "alice")) ;; roles
(def bob   (role "bob"))

(def ttt-close (dsl ;; auxiliary spec
  (par (-## alice bob)
       (-## bob alice))))
(def ttt (dsl ;; main spec
  (fix :X
    [(--> alice bob Long)
     (alt (ins ttt-close)
          [(--> bob alice Long)
           (alt (ins ttt-close)
                (fix :X))])])))
```

**Fig. 5.** Discourje specification of Tic-Tac-Toe

```
10 (def m (moni (spec ttt)))
11 (def a->b (chan 1 alice bob m)) (def b<-a a->b)
12 (def b->a (chan 1 bob alice m)) (def a<-b b->a)
```

**Fig. 6.** Changes to Fig. 3 to monitor Alice and Bob against the specification in Fig. 5

To monitor the implementations of Alice and Bob against this specification, first, we need to load library `discourje.core.async` instead of `clojure.core.async` (implicitly loaded in Fig. 3). All other code modifications are shown in Fig. 6: on line 10, the specification is evaluated to an internal form (**`spec`**) and wrapped in a new monitor (**`moni`**), while on lines 11–12, we associate the intended sender, receiver, and monitor with the channels. *No other changes are needed:* notably, the code for Alice (Fig. 3, lines 14–24) and Bob (lines 25–35) is unaffected; Discourje is non-invasive to start using. Running the monitor alongside the implementation guarantees safety: if a non-compliant channel action were to be attempted, the monitor prevents it from happening and throws an exception.

The implementation in Fig 3 can *indeed* violate the specification in Fig. 5: the specification states channels are allowed to be closed only *after* (the receive of) the previous communication is done, but in the implementation, Alice or Bob could attempt to close already *before*. In our artifact, we have a solution where we mix channels with *barrier synchronization* from the standard `java.util.concurrent` library (readily usable in Clojure), to let Alice and Bob first await each other and then close. Thus, channel-based programming abstractions monitored through Discourje can be mixed seamlessly with other concurrency libraries, which happens regularly in message passing programs [46,48].

**Advanced Discourje: common patterns.** Discourje specifications of common patterns of communication are shown in Fig. 7; they make use of Discourje's *role indexing* and finite *repetition* (**`rep`**) features.

Imagine we have a sequence of worker threads, organized in a pipeline (i.e., the $i$-th worker receives from its predecessor, $i-1$, and sends to it successor, $i+1$). Lines 1–2 define the specification of a communication from a worker to its successor. Intuitively, `succ` is a function that maps three parameters to a specification. For instance, `(`**`ins`**` succ bob 5 Turn)` inserts `(`**`-->`**` (bob 5) (bob 6) Turn)`, where `(bob 5)` and `(bob 6)` are indexed roles. We note that every role created with **`role`** allows indexing (with arbitrary types), and that specifications can be

```
1 (def succ (dsl :w :i :t
2   (--> (:w :i) (:w (inc :i)) t)))
3
4 (def pipe (dsl :w :k :t
5   (rep seq [:i (range (dec :k))]
6     (ins succ :w :i :t))))
7
8 (def ring (dsl :w :k :t
9   [(ins pipe :w :k :t)
10    (--> (:w (dec :k)) (:w 0) :t)]))
11 (def one-one-one (dsl :m :w :k :t :u
12   (rep alt [:i (range :k)]
13     [(--> :m (:w :i) :t)
14      (--> (:w :i) :m :u)]))
15
16 (def one-all-one (dsl :m :w :k :t :u
17   (rep par [:i (range :k)]
18     [(--> :m (:w :i) :t)
19      (--> (:w :i) :m :u)]))
```

**Fig. 7.** Discourje specification of common patterns parametrized by roles (`:w`), indices (`:i`), and/or types (`:t`). We also note that *any* Clojure function can be used in specifications (e.g., `inc`, to manipulate indices).

Lines 4–6 define the specification of a pipeline communication pattern; it states that specification `(ins succ :w :i :t)` is repeated for each value `:i` from `0` to `k-1`, and the iterations are composed sequentially (`seq`). Lines 8–10 extend the pipeline to a ring, where the last worker also communicates with the first.

Lines 11–14 define the specification of a communication from a "master" to *one* of $k$ workers, and back. Similarly, lines 16–19 define the specification of a communication from a master to *all* of $k$ workers, and back. In these specifications, loop iterations are composed alternatively (`alt`) and in parallel (`par`).

### 3 Design

**Implementation calculus.** To formalize our verification problem, we first define a calculus to model Clojure implementations. Let $\ell$ range over heap locations, $x$ over variables, $v$ over values, and $I$ over implementations. The calculus is generated by the following grammar:

$$v ::= \mathsf{nil} \mid \ell \mid \mathsf{fn}\ x\ I \mid \mathsf{true} \mid \mathsf{false} \mid 0 \mid 1 \mid 2 \mid \ldots$$

$$I ::= v \mid I_1\ I_2 \mid x \mid \mathsf{def}\ x\ I \mid \mathsf{let}\ x\ I_1\ I_2 \mid \mathsf{loop}\ x\ I_1\ I_2 \mid \mathsf{recur}\ I \mid$$
$$\mathsf{if}\ I_1\ I_2\ I_3 \mid I_1 \cdot I_2 \mid \mathsf{send}\ I_1\ I_2 \mid \mathsf{recv}\ I \mid \mathsf{close}\ I \mid \mathsf{chan}\ I \mid I_1 \parallel I_2$$

Calculus notation corresponds closely with Clojure notation (Fig. 4), with the exception of application ($I_1\ I_2$), sequencing ($I_1 \cdot I_2$), and threading ($I_1 \parallel I_2$).

The operational semantics of the calculus is defined in terms of labeled reductions of triples $(I, \mathcal{E}, \mathcal{H})$: $I$ is an implementation, $\mathcal{E}$ is a global environment (from variables to values), and $\mathcal{H}$ is a heap (from heap locations to channel states). Channel states are represented as pairs $(\boldsymbol{w}, n)$, where $\boldsymbol{w}$ is a list of values (messages in transit, from left to right), and $n$ the buffer size. Labels, ranged over by $\alpha$, are of the form $\ell!v$ (send), $\ell?v$ (receive), $\ell\#$ (close), and $\tau$ (anything else; we verify only channel actions). The reduction rules are shown in Fig. 8.

Rule [I-Ctxt] executes the first step of implementation $I$ in context $\mathcal{C}$: it first substitutes $I$ for $\square$ in $\mathcal{C}$ (notation: $\mathcal{C}[I]$), and then executes the first step.

$$\frac{(I,\mathcal{E},\mathcal{H}) \xrightarrow{\alpha} (I',\mathcal{E}',\mathcal{H}')}{(\mathcal{C}[I],\mathcal{E},\mathcal{H}) \xrightarrow{\alpha} (\mathcal{C}[I'],\mathcal{E}',\mathcal{H}')}\text{[I-Ctxt]} \qquad \frac{I[v/x] \xrightarrow{\alpha} I'}{((\mathsf{fn}\ x\ I)\ v,\mathcal{E},\mathcal{H}) \xrightarrow{\alpha} (I',\mathcal{E},\mathcal{H})}\text{[I-App]}$$

$$\frac{\mathcal{E}(x) = v}{(x,\mathcal{E},\mathcal{H}) \xrightarrow{\tau} (v,\mathcal{E},\mathcal{H})}\text{[I-Var]} \qquad \frac{}{(\mathsf{def}\ x\ v,\mathcal{E},\mathcal{H}) \xrightarrow{\tau} (\mathsf{nil},\mathcal{E}[x \mapsto v],\mathcal{H})}\text{[I-Def]}$$

$$\frac{I[v/x] \xrightarrow{\alpha} I'}{(\mathsf{let}\ x\ v\ I,\mathcal{E},\mathcal{H}) \xrightarrow{\alpha} (I',\mathcal{E},\mathcal{H})}\text{[I-Let]} \qquad \frac{I[v/x][(\mathsf{fn}\ x_{\mathrm{r}}\ (\mathsf{loop}\ x\ x_{\mathrm{r}}\ I))/\mathsf{recur}\,] \xrightarrow{\alpha} I'}{(\mathsf{loop}\ x\ v\ I,\mathcal{E},\mathcal{H}) \xrightarrow{\alpha} (I',\mathcal{E},\mathcal{H})}\text{[I-Loop]}$$

$$\frac{v \in \{\mathsf{true},\mathsf{false}\} \qquad (I_v,\mathcal{E},\mathcal{H}) \xrightarrow{\alpha} (I'_v,\mathcal{E},\mathcal{H})}{(\mathsf{if}\ v\ I_{\mathsf{true}}\ I_{\mathsf{false}},\mathcal{E},\mathcal{H}) \xrightarrow{\alpha} (I'_v,\mathcal{E},\mathcal{H})}\text{[I-If]} \qquad \frac{(I,\mathcal{E},\mathcal{H}) \xrightarrow{\alpha} (I',\mathcal{E},\mathcal{H})}{(v \cdot I,\mathcal{E},\mathcal{H}) \xrightarrow{\alpha} (I',\mathcal{E},\mathcal{H})}\text{[I-Seq]}$$

$$\frac{\mathcal{H}(\ell) = (\boldsymbol{w}, n)\ \textbf{and}\ |\boldsymbol{w}| < n}{(\mathsf{send}\ \ell\ v,\mathcal{E},\mathcal{H}) \xrightarrow{\ell!v} (\mathsf{nil},\mathcal{E},\mathcal{H}[\ell \mapsto (v{\cdot}\boldsymbol{w}, n)])}\text{[I-Send]} \qquad \frac{\mathcal{H}(\ell) = (\boldsymbol{w}{\cdot}v, n)}{(\mathsf{recv}\ \ell,\mathcal{E},\mathcal{H}) \xrightarrow{\ell?v} (v,\mathcal{E},\mathcal{H}[\ell \mapsto (\boldsymbol{w}, n)])}\text{[I-Recv]}$$

$$\frac{\mathcal{H}(\ell) = (\boldsymbol{w}, n)\ \textbf{and}\ n > 0}{(\mathsf{close}\ \ell,\mathcal{E},\mathcal{H}) \xrightarrow{\ell\#} (\mathsf{nil},\mathcal{E},\mathcal{H}[\ell \mapsto (\boldsymbol{w}, 0)])}\text{[I-Close]} \qquad \frac{\mathcal{H}(\ell) = \bot\ \textbf{and}\ [\![v]\!] > 0}{(\mathsf{chan}\ v,\mathcal{E},\mathcal{H}) \xrightarrow{\tau} (\ell,\mathcal{E},\mathcal{H}[\ell \mapsto (\epsilon, [\![v]\!])])}\text{[I-Chan]}$$

**Fig. 8.** Operational semantics of the implementation calculus

Contexts are generated by the following grammar:

$$\begin{aligned}\mathcal{C} ::= \Box \mid \mathcal{C}\ I \mid (\mathsf{fn}\ x\ I)\ \mathcal{C} \mid \mathsf{def}\ x\ \mathcal{C} \mid \mathsf{let}\ x\ \mathcal{C}\ I \mid \mathsf{loop}\ x\ \mathcal{C}\ I \mid \mathsf{if}\ \mathcal{C}\ I_{\mathrm{t}}\ I_{\mathrm{f}} \mid \mathcal{C} \cdot I \mid \\ \mathsf{send}\ \mathcal{C}\ I \mid \mathsf{send}\ \ell\ \mathcal{C} \mid \mathsf{recv}\ \mathcal{C} \mid \mathsf{close}\ \mathcal{C} \mid \mathsf{chan}\ \mathcal{C} \mid \mathcal{C} \parallel I \mid I \parallel \mathcal{C}\end{aligned}$$

Rule [I-App] executes the first step of a function: it first substitutes value $v$ for variable $x$ in body $I$ (notation: $I[v/x]$), and then executes the first step. Rule [I-Var] executes a read in the global environment. Rule [I-Def] executes a write to the global environment (notation: $\mathcal{E}[x \mapsto v]$). Rule [I-Let] executes the first step of a let binder, similar to rule [I-App]. Rule [I-Loop] executes the first step of a loop: it first substitutes value $v$ for variable $x$ (the loop parameter) in body $I$, then substitutes the loop itself (wrapped in a function to rebind $x$ in the loop's next iteration) for recur, and then executes the first step. Rule [I-If] executes the first step of a branch of a conditional, if the condition is boolean. Rule [I-Seq] executes the first step of the suffix of a sequence, after the prefix has been executed using rule [I-Ctxt]. Rule [I-Send] executes the send through a channel, if that channel exists and is not full. Rule [I-Recv] executes the receive through a channel, if that channel exists and is not empty. Rule [I-Close] executes the close of a channel, if that channel exists and is not yet closed. Rule [I-Chan] executes the creation of a new channel.

**Specification calculus.** Next, we define a calculus to model Discourje specifications. Let $p, q$ range over roles, $f$ over boolean functions (from the implementation calculus), $n, m$ over number expressions (from the implementation calculus),

$$\frac{}{1\downarrow}\text{[S}\downarrow\text{-One]} \quad \frac{S_{i\in\{1,2\}}\downarrow}{S_1+S_2\downarrow}\text{[S}\downarrow\text{-Alt]} \quad \frac{S_1\downarrow \textbf{ and } S_2\downarrow}{S_1\cdot S_2\downarrow}\text{[S}\downarrow\text{-Seq]} \quad \frac{S_1\downarrow \textbf{ and } S_2\downarrow}{S_1\parallel S_2\downarrow}\text{[S}\downarrow\text{-Par]}$$

**Fig. 9.** Operational semantics of the specification calculus (termination)

$$\frac{(f\ v,\emptyset,\emptyset)\xrightarrow{\tau}{}^{*}(\mathsf{true},\emptyset,\emptyset)}{p[n]\twoheadrightarrow q[m]:f \xrightarrow{p[n]q[m]!v} p[n]q[m]?v \xrightarrow{p[n]q[m]?v} 1}\text{[S-Com]} \quad \frac{S=p[n]\not\twoheadrightarrow q[m]}{S\xrightarrow{p[n]q[m]\#}1}\text{[S-Cls]}$$

$$\frac{S_{i\in\{1,2\}}\xrightarrow{\beta}S'}{S_1+S_2\xrightarrow{\beta}S'}\text{[S-Alt]} \quad \frac{S_1\xrightarrow{\beta}S_1'}{S_1\cdot S_2\xrightarrow{\beta}S_1'\cdot S_2}\text{[S-Seq1]} \quad \frac{S_1\downarrow \textbf{ and } S_2\xrightarrow{\beta}S_2'}{S_1\cdot S_2\xrightarrow{\beta}S_2'}\text{[S-Seq2]}$$

$$\frac{S[\text{fix}\,X\,S/X]\xrightarrow{\beta}S'}{\text{fix}\,X\,S\xrightarrow{\beta}S'}\text{[S-Rec]} \quad \frac{S_1\xrightarrow{\beta}S_1'}{S_1\parallel S_2\xrightarrow{\beta}S_1'\parallel S_2}\text{[S-Par1]} \quad \frac{S_2\xrightarrow{\beta}S_2'}{S_1\parallel S_2\xrightarrow{\beta}S_1\parallel S_2'}\text{[S-Par2]}$$

$$\frac{S[n/x]\otimes(\ldots\otimes(S[n'{-}1/x]\otimes S[n'/x]))\xrightarrow{\beta}S'}{\bigotimes^{\otimes}_{n\le x\le n'}S\xrightarrow{\beta}S'}\text{[S-Rep]}$$

**Fig. 10.** Operational semantics of the specification calculus (reduction)

and $\otimes$ over $\{+,\cdot,\parallel\}$. The calculus is generated by the following grammar:

$$S ::= \boxed{1} \mid p[n]\twoheadrightarrow q[m]:f \mid \boxed{p[n]q[m]?v} \mid p[n]\not\twoheadrightarrow q[m] \mid S_1+S_2 \mid S_1\cdot S_2 \mid S_1\parallel S_2 \mid \text{fix}\,X\,S \mid X \mid \bigotimes^{\otimes}_{n\le x\le n'}S$$

Calculus notation corresponds with Discourje notation (Sect. 2): $p[n]\twoheadrightarrow q[m]:f$ specifies communication of a value that satisfies $f$ from $p[n]$ to $p[m]$; $p[n]\not\twoheadrightarrow q[m]$ specifies closing of the channel from $p[n]$ to $q[m]$; $S_1\otimes S_2$ specifies the alternative, sequential, and parallel composition of $S_1$ and $S_2$; fix $X\,S$ and $X$ specify recursion; and $\bigotimes^{\otimes}_{n\le x\le n'}S$ specifies repetition of $S$ for every value $x$ between $n$ and $n'$, where iterations are composed using $\otimes$. "Boxed" specifications (1 and $p[n]q[m]?v$; the box is not part of the syntax) are *auxiliary* in the sense they are used in defining the operational semantics (below), but they are not written directly in specifications by programmers: 1 specifies a skip; $p[n]q[m]?v$ specifies a receive of $v$ by $q[m]$, previously sent by $p[n]$.

The operational semantics of the calculus is defined in terms of termination predicate $\downarrow$ and labeled reduction relation $\rightarrow$. Labels, ranged over by $\beta$, are of the form $p[n]q[m]!v$ (send), $p[n]q[m]?v$ (receive), and $p[n]q[m]\#$ (close). The termination and reduction rules are shown in Figs. 9–10. (This operational semantics coincides with Basic Process Algebra [22], plus free merge, recursion, and repetition.) Rule [S-Com] induces two reductions (first a send, then a receive), via auxiliary specification $p[n]q[m]?v$. We note that the specification calculus has no $\tau$-reductions (which are not monitored; we verify only channel actions). We also note that it can express some, but not all, context-free languages: it can count (using $\bigotimes$), but it cannot encode a stack.

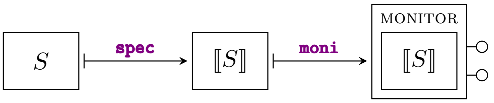

**Fig. 11.** DSL workflow

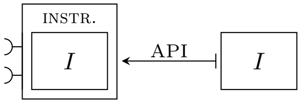

**Fig. 12.** API workflow

**Inclusion relation.** Finally, we define a relation to decide if the behavior of an implementation $I$ is included in the behavior of a specification $S$.

First, let $\dagger$ range over functions from heap locations to sender–receiver pairs; informally, $\dagger$ establishes a correspondence between channel references in the implementation (characterized by their heap locations) and channel references in the specification (characterized by the roles that use them as sender/receiver).

Next, let $\rightarrow_I \subseteq \rightarrow$. We call $\rightarrow_I$ an *execution* of $I$ if it satisfies these conditions:

- $(I, \emptyset, \emptyset) \xrightarrow{\alpha}_I (\hat{I}', \mathcal{E}', \mathcal{H}')$;
- if $(\hat{I}, \mathcal{E}, \mathcal{H}) \xrightarrow{\alpha}_I (\hat{I}', \mathcal{E}', \mathcal{H}')$, then $(\hat{I}', \mathcal{E}', \mathcal{H}') \xrightarrow{\alpha'}_I (\hat{I}'', \mathcal{E}'', \mathcal{H}'')$ or $\hat{I}'$ is a value;
- if $(\hat{I}, \mathcal{E}, \mathcal{H}) \xrightarrow{\alpha_1}_I (\hat{I}'_1, \mathcal{E}'_1, \mathcal{H}'_1)$ and $(\hat{I}, \mathcal{E}, \mathcal{H}) \xrightarrow{\alpha_2}_I (\hat{I}'_2, \mathcal{E}'_2, \mathcal{H}'_2)$, then $\alpha_1 = \alpha_2$ and $(\hat{I}'_1, \mathcal{E}'_1, \mathcal{H}'_1) = (\hat{I}'_2, \mathcal{E}'_2, \mathcal{H}'_2)$.

Finally, a $(\dagger, \rightarrow_I)$-*simulation* $\mathbf{R}$ is a binary relation such that if $(\hat{I}, \mathcal{E}, \mathcal{H}) \xrightarrow{\alpha}_I (\hat{I}', \mathcal{E}', \mathcal{H}')$ and $(\hat{I}, \mathcal{E}, \mathcal{H})\ \mathbf{R}\ S$, then for some $S'$:

- if $\alpha \in \{\ell!v, \ell?v, \ell\#\}$ for some $\ell, v$, then $S \xrightarrow{\alpha[\dagger(\ell)/\ell]} S'$ and $(\hat{I}', \mathcal{E}', \mathcal{H}')\ \mathbf{R}\ S'$;
- if $\alpha = \tau$, then $(\hat{I}', \mathcal{E}', \mathcal{H}')\ \mathbf{R}\ S$.

In words, $(\hat{I}, \mathcal{E}, \mathcal{H})\ \mathbf{R}\ S$ iff whenever $\hat{I}$ can reduce to $\hat{I}'$, $S$ can reduce accordingly to $S'$ (and $\hat{I}'$ and $S'$ are again related by $\mathbf{R}$), up to $\tau$-reductions ($\mathbf{R}$ is weak [24]).

Implementation $I$ is *safe* relative to specification $S$, denoted as $I \preceq S$, if for every execution $\rightarrow_I$ of $I$, there is a $(\dagger, \rightarrow_I)$-simulation $\mathbf{R}$ such that $(I, \emptyset, \emptyset)\ \mathbf{R}\ S$.

### 4 Implementation

**The DSL.** The DSL consists of: Clojure macros to write specifications (cf. syntax of the specification calculus; Sect. 3); Clojure data structures to represent specifications as state machines (cf. operational semantics of the specification calculus); Clojure functions to instantiate these data structures and construct monitors. The workflow is shown in Fig. 11: first, the programmer writes a specification $S$ using the macros; then, at run-time, function **`spec`** is applied to $S$ to expand and evaluate the macros to a data structure $[\![S]\!]$; then, function **`moni`** is applied to $[\![S]\!]$ to construct a monitor.

Essentially, the monitor provides two operations, depicted as "lollipops" in Fig. 11: *checking* if a given channel action $\alpha$ is allowed by $[\![S]\!]$ (formally: $S \xrightarrow{\alpha} S'$ for some $S'$), and subsequently *updating* $[\![S]\!]$ to its successor. In this way, effectively, the monitor incrementally builds a formal simulation to ensure safety (Sect. 3, page 275). We note that checking/updating is protected by lock-free synchronization (compare-and-set): an $\alpha$ reduction happens only if it was already checked if $\alpha$ is allowed, *and* the state has not yet been updated after that check.

**The API.** The API consists of Clojure functions that act as proxies for Clojure's own functions to send, receive, close channels, and construct channels. The workflow is shown in Fig. 12: first, the programmer writes an implementation $I$ using Clojure's own functions; then, by loading library `discourje.core.async` instead of `clojure.core.async`, the programmer adds instrumentation to the implementation that allows channel actions to be monitored. As the signatures of Discourje's send, receive, and close functions are identical to Clojure's, adding instrumentation in this way is non-invasive and nearly effortless; the only changes needed, pertain to channel creation (Sect. 2, Fig. 6), since we require the programmer also to specify which roles will use the channel and associate a monitor (this is the practical embodiment of function † in Sect. 3, page 275).[4]

Discourje's send function works as follows. When invoked, first, it waits until the underlying channel $c$ is not full (recall channels in Clojure are bounded and blocking). Then, at time $t_1$, it calls the monitor associated with $c$ to check if the send is allowed. If yes, at time $t_2$, it calls the monitor to update accordingly and the "actual send" happens through $c$; if no, only an exception is thrown. If, between $t_1$ and $t_2$, multiple threads call the monitor to update, only one will succeed; the others need to retry from the start. Discourje's receive and close functions work similarly. In this way, Discourje detects safety violations in a way that is both sound (if an exception is thrown, the violating action really was not allowed) and complete (if no exception is thrown, all actions were really allowed).

**Extensions.** We implemented a number of extensions to the basic framework:

- **Multi-cast:** Adding to Clojure's send, receive, and close functions, the API also contains a multi-cast function to send the same value through $n{>}1$ channels, along with special monitoring support in the DSL (more efficient than monitoring individual communications). Also, the API contains a "multi-receive" function that optionally synchronizes all receivers of a multi-cast.
- **Java interoperability:** Clojure compiles to Java bytecode and runs on the JVM; this enabled us to extend Discourje to Java. Specifically, we wrote a thin Java wrapper around Discourje, so Java programmers can easily construct and use Discourje channels, write specifications, and have them monitored from inside their Java programs, regardless of the threading mechanism (e.g., classical Java threads, thread pools, and parallel streams can be used).

### 5 Evaluation

**General setup.** We developed Discourje for two primary usage types:

[4] We currently support the following main channel operations of `clojure.core.async`: sending, receiving, and closing. Discourje works out-of-the-box for all Clojure programs, except those that use unsupported `clojure.core.async` features; mixing Discourje with other concurrency libraries is fine (Sect. 2).

An interesting next step is to also support `clojure.core.async`'s *transducers* (operations on data-in-transit): to our knowledge, no existing work on MPST supports transducers, so supporting those requires significant new theoretical work.

A. as a *testing/debugging tool* for concurrent programs in development, to reliably find/diagnose communication-related concurrency bugs;
B. as a *fail-safe mechanism* for concurrent programs in production, to prevent propagation of spurious results caused by concurrency bugs to end-users (i.e., it is better to throw a runtime error, cf. `ArrayIndexOutOfBoundsException`.)

A key factor that determines Discourje's fitness for purpose is *overhead*. We therefore conducted two kinds of benchmarks: microbenchmarks to study the *scalability* of Discourje and whole-program benchmarks to study the *slowdown* it inflicts relative to unmonitored code.

We used two different hardware configurations to run our benchmarks: VM2 is an instance of the TACAS'20 Artifact Evaluation Virtual Machine for VirtualBox, configured with 2 virtual cores and 8 GB of virtual memory; LISA is a high-end machine with 16 physical cores (Intel Xeon 6130 processor; hyper-threading disabled) and 96 GB of physical memory (far more than needed for our benchmarks). We hosted VM2 on a machine with 4 physical cores (Intel Core i7-8569U; hyper-threading enabled) and 16 GB of physical memory.

**Microbenchmarks.** In the microbenchmarks, we studied Discourje's scalability under extreme circumstances where threads perform *only* sends/receives and no real computations; this is the worst-case scenario for the lock-free algorithm to synchronize monitor access, as it gives rise to maximal thread contention.

We considered three specifications to investigate the core features/operators offered by the Discourje DSL in isolation, using our built-in common patterns (Fig. 7): `ring` for sequential composition, `one-one-one` (OOO) for alternative composition, and `one-all-one` (OAO) for parallel composition. Each pattern was recursively repeated (i.e., wrapped in `(fix :X [... (fix :X)])`). For Ring and OAO, a *round* consists of 1000 repetitions; for OOO, a round consists of $1000{\cdot}n$ repetitions, where $n$ is the number of worker threads.

For each implementation $I \in \{\text{Ring}, \text{OOO}, \text{OAO}\}$ with $n \in \{2, 4, 6, 8, 10, 12, 14, 16\}$ worker threads,[5] we recorded the mean round latency $\mu_n^I$ in eight hours of execution on LISA, the standard deviation $\sigma_n^I$, and the coefficient of variation $c_n^I{=}\frac{\mu_n^I}{\sigma_n^I}$. We found $c_n^I \leq 6\%$ for all $I$ and $n$, except $c_6^{\text{OOO}} = 14\%$ and $c_8^{\text{OOO}} = 8\%$.

As a measure of scalability, we computed normalized means $|\mu_n^I| = \frac{\mu_n^I}{0.5 \cdot n \cdot \mu_2^I}$: this metric is a dimensionless number that indicates the extent to which implementations scale linearly in the number of worker threads, relative to $n = 2$. For instance, if $|\mu_{16}^I| = 1$, $I$ with 16 workers threads is exactly $8\times$ as slow as $I$ with 2 worker threads; this is reasonable, because the worker threads perform $8\times$ more sends and receives in each round (due to the adversarial microbenchmark conditions, the sends and receives are effectively linearized by the monitor, which can check and update at most one channel action at a time).

The normalized means are shown in Fig. 13; our raw data (including standard deviations) are included in our artifact. We summarize the findings:

[5] For Ring, the total number of threads is $n$; for OOO and OAO, the total number of threads is $n{+}1$ (the master thread).

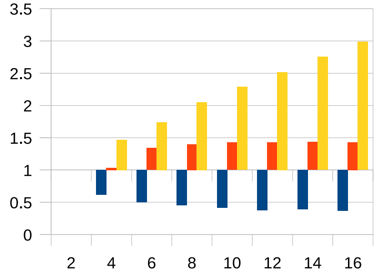

**Fig. 13.** Microbenchmarks on LISA: Ring (blue), OOO (red), and OAO (yellow); number of threads (x-axis) vs. scalability relative to $n = 2$ (y-axis)

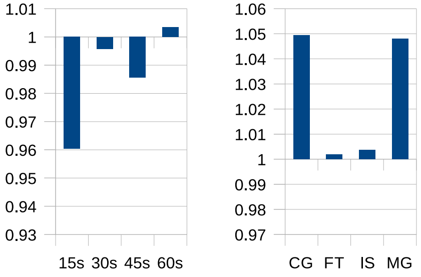

**Fig. 14.** Whole-program benchmarks on VM2: Chess (left) and NPB (right); play time (x-axis, left) and program (x-axis, right) vs. monitoring slowdown (y-axis)

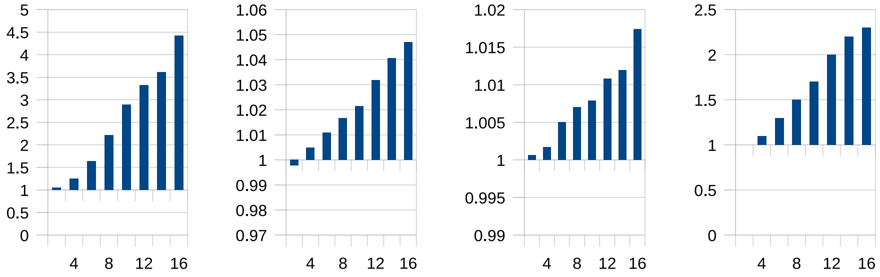

**Fig. 15.** Whole-program benchmarks on LISA: CG, FT, IS, and MG (from left to right); number of threads (x-axis) vs. monitoring slowdown (y-axis)

- Ring (blue) scales sub-linearly. This is because at any point in time, only one worker thread contends for monitor access (the current receiver or sender; the others are blocked, waiting for incoming channels to become non-empty).
- OOO (red) scales linearly, stabilizing around a constant factor of 1.4. This is because the number of branches in the monitor's internal state machine grows linearly in the number of worker threads. Thus, the cost of using the monitor grows proportionately, but the factor is constant.
- OAO (yellow) scales super-linearly, getting progressively worse as the number of worker threads increases. This is because all worker threads contend for monitor access all the time, *and* the number of branches in the monitor's state machine increases linearly.

To conclude, Ring (which exercises sequential composition) enjoys excellent scalability, while OOO (which exercises alternative composition) enjoys decent scalability, even under the adversarial microbenchmark conditions. Scalability of OAO (parallel composition) can be improved; we discuss one avenue in Sect. 7.

**Whole-program benchmarks.** In our whole-program benchmarks, we studied Discourje's possible slowdown in five real(istic)/existing concurrent programs:

- **Chess:** Simulates a game of chess between two player threads.
- **Conjugate Gradient (CG-$n$):** Computes an estimate of the largest eigenvalue of a symmetric positive definite sparse matrix with a random pattern of nonzeros, using the conjugate gradient algorithm, with $n$ worker threads.
- **Fourier Transform (FT-$n$):** Computes the solution of a partial differential equation, using the forward and inverse Fast Fourier Transform algorithm, with $2{\cdot}n$ worker threads.
- **Integer Sort (IS-$n$):** Computes a sorted list of uniformly distributed integer keys, using histogram-based integer sorting, with $n$ worker threads.
- **Multi-Grid (MG-$n$):** Computes an approximate solution $u$ to the discrete Poisson problem $\nabla^2 u = v$, using the V-cycle multigrid algorithm, with $4{\cdot}n$ worker threads.

For Chess, we used Clojure code similar to threads Alice and Bob in Tic-Tac-Toe (Fig. 3), combined with invocations of the open source chess engine Stockfish 10 (https://stockfishchess.org) to compute moves. For CG, FT, IS, and MG, we adapted existing Java implementations from the *NAS Parallel Benchmarks* (NPB) [23] suite, which consists of computational fluid dynamics kernels, by taking advantage of our Java interoperability wrapper (Sect. 4) to replace the monitor-based synchronization used in the original versions.

We also wrote specifications for these implementations in the Discourje DSL. For Chess, the specification is the same as the Tic-Tac-Toe specification (Sect. 2); for CG, FT, IS, and MG, the specifications consist of recursively repeated choices among various instances of the `one-all-one` pattern (each of which involves different subsets of worker threads and message types); the key difference between the specifications, then, is the *frequency* in which repetitions occur.

We recorded execution times of each of the implementations without and with monitoring enabled, using existing/standardized workloads. For Chess, the workload is controlled by the total amount of time each player has to compute its moves during the entire game; we used the four smallest such workloads supported by the open source chess server Lichess (https://lichess.org), namely $\{15, 30, 45, 60\}$ seconds, and we limited games to a maximum of 40 turns per player (*UltraBullet chess*).[6] For CG, FT, IS, and MG, the workload is controlled by the input size; we used the standardized inputs that are predefined by NPB.

We ran Chess on VM2; we ran CG-$n$, FT-$n$, IS-$n$, and MG-$n$ on VM2 for $n = 2$ and on LISA for $n \in \{2, 4, 6, 8, 10, 12, 14, 16\}$. We repeated each of the runs 50 times to smooth out variability; the resulting coefficients of variation are below 5% for CG, FT, IS, and MG, and between 19%–22% for Chess (because moves are not computed deterministically, which affects the number of turns per game). As a measure of slowdown, we computed normalized means of execution times with monitoring, $\mu_{\mathrm{w}}$, against those without monitoring, $\mu_{\mathrm{wo}}$ (i.e., $\frac{\mu_{\mathrm{w}}}{\mu_{\mathrm{wo}}}$): this

[6] We allow concurrent "ponder" computations during opponents' turns.

metric is a dimensionless number that indicates the factor by which monitoring slows down the implementation.

The normalized means are shown in Figs. 14-15; the raw data (including standard deviations) are included in our artifact. We summarize the findings:

- For Chess, for three workloads, slowdowns are $<1$. As the number of instructions per channel action is, objectively, higher with monitoring than without, we suspect these observed *speedups* might be an artifact of the variability in the measurements. That said, the general trend suggests both usage types of Discourje (page 277) are very well possible for Chess.
- For FT and IS, the slowdowns are low: less than 5% and 2% respectively. This seems low enough not only for Discourje's usage type A (testing/debugging in development), but even usage type B (fail-safe mechanism in production).
- For CG and MG, the slowdowns are higher: less than $5\times$ and $2.5\times$ respectively. Although this might be too much for Discourje's usage type B, it seems low enough for usage type A (cf. the industrial-strength Valgrind tool for memory debugging [35], which typically inflicts a $\geq 10\times$ slowdown).
  The difference in performance between {FT, IS} and {CG, MG} may be explained by the fact the latter are more communication-intensive than the former, so the overhead of monitoring communications is more pronounced.
- For CG, FT, IS, and MG, the slowdowns grow only linearly as the number of threads increases. This shows that the super-linear scalability we observed under the adversarial microbenchmark conditions for the `one-all-one` pattern, does not manifest in these real programs.

To conclude, we believe it is encouraging to see that *even* (extended versions of) the specification that scaled poorest in our microbenchmarks, can give well enough performance in real concurrent programs for both usage types A and B.

### 6 Related Work

Expressiveness issues of multiparty session types (MPST) have received some attention, but efforts have primarily been geared towards adding more advanced features (e.g., time [5,36], security [7,8,9,13], and parametrisation [14,20,39]); in contrast, restrictions on the usage of core features like choice and interleaving have remained, even though they limit MPST's applicability in practice (e.g., our Tic-Tac-Toe specification cannot be expressed; Fig. 5). Recently, work has been done to improve MPST's expressiveness in this regard using static techniques [31], but our specification language in this paper is still more expressive.

Closest to our work, then, are hybrid MPST approaches that combine static type-checking with a form of distributed runtime monitoring and/or assertion checking [3,4,19,36,37]. In contrast to this paper, however, these dynamic techniques still rely on projection, which limits expressiveness (Sect. 1); none of the specifications in this paper are supported.

Projection-free MPST has also been explored by López et al. [34,43]. Their idea is to specify MPI communication protocols in an MPI-tailored DSL, inspired by MPST, and verify the implementation against the specification using deductive verification tools (VCC [18] and Why3 [21]). However, this approach does not support push-button verification: considerable manual effort is required. In contrast, our approach is fully automated.

We are aware of only two other works that use formal techniques to reason about Clojure programs: Bonnaire-Sergeant et al. [6] formalized the optional type system for Clojure and proved soundness, while Pinzaru et al. [41] developed a translation from Clojure to Boogie [2] to verify Clojure programs annotated with pre/post-conditions. Ours is the first paper that targets concurrency in Clojure.

Verification of shared-memory concurrency with channels has received attention in the context of Go [40,32,33,45]. However, emphasis in these works is on checking deadlock-freedom, liveness, and generic safety properties, while we focus on program-specific protocol compliance. Castro et al. [14] also consider protocol compliance, but their specification language (of global types) is less expressive than ours and does not support this paper's examples.

### 7 Conclusion

We presented Discourje: a runtime verification framework for channel-based communication protocols in Clojure. Discourje is based on a projection-free interpretation of multiparty session types, trading static type-checking for dynamic runtime monitoring to alleviate expressiveness issues. A key design principle of Discourje has been ergonomics: we aim to make Discourje's use as comfortable as possible. Specifically, programmers can decide to start using Discourje at any stage of development (and doing so requires little effort); Discourje is itself implemented in Clojure (so no need to use a different IDE, learn completely new syntax, or install special compilers); and Discourje can be used seamlessly alongside other concurrency libraries. The framework has a formal foundation, and benchmarks indicate that monitoring overhead can be less than 5% for real/existing concurrent programs. This makes Discourje suitable both as a testing/debugging tool in development, and as a fail-safe mechanism in production.

We list two interesting avenues for future work. First, we want to refine our lock-free synchronization algorithm to enhance the way parallel composition is handled. Second, a much more profound extension pertains to *feedback* and *recovery*. Specifically, we want to explore the idea that whenever a monitor detects a violation, instead of throwing an exception, it should simply *delay* the violating action as a corrective measure, in an attempt to steer the implementation toward safe behavior. When done naively, such delays can easily yield deadlocks, so our plan is to combine this with runtime model-checking/reachability analysis to check if *eventually*, the violating action is allowed (if yes, delay; if no, throw).

*Acknowledgments.* Funded by the Netherlands Organisation of Scientific Research (NWO): 016.Veni.192.103. This work was carried out on the Dutch national e-infrastructure with the support of SURF Cooperative.
